# A novel joint index based on peripheral blood CD4+/CD8+ T cell ratio, albumin level, and monocyte count to determine the severity of major depressive disorder

**DOI:** 10.1186/s12888-022-03911-5

**Published:** 2022-04-08

**Authors:** Dechun Zhou, Hongfeng Yu, Hongfeng Yao, Shijin Yuan, Yan Xia, Lei Huang, Yuedi Shen, Jun Zhang, Wei Chen

**Affiliations:** 1 Department of Psychiatry, Sir Run Run Shaw Hospital, School of Medicine, Zhejiang University, 3 Qingchun East Road, Hangzhou, 310016 China; 2 Department of Clinical Laboratory, Ciwu branch of Zhuji Peoples Hospital, Shaoxing, 311815 China; 3 Department of Clinical Laboratory, Zhuji Peoples Hospital, Shaoxing, 311899 China; 4 Department of Clinical Laboratory, Sir Run Run Shaw Hospital, School of Medicine, Zhejiang University, 3 Qingchun East Road, Jianggan District, Hangzhou, 310016 China; 5 Department of Diagnostics, School of Medicine, Hangzhou Normal University, Hangzhou, 311121 China; 6 Department of Psychology and Behavioral Sciences, Zhejiang University, Hangzhou, 310012 China; 7 Key Laboratory of Medical Neurobiology of Zhejiang Province, Hangzhou, 310016 China

**Keywords:** Major depressive disorder, Peripheral blood laboratory investigation, Biomarker, Joint index

## Abstract

**Background:**

Inflammation and immune status are correlated with the severity of major depressive disorder (MDD).The purpose of this study was to establish an optimization model of peripheral blood parameters to predict the severity of MDD.

**Methods:**

MDD severity in the training and validation cohorts (*n* = 99 and 97) was classified using the Hamilton Depression Scale, Thirty-eight healthy individuals as controls. Significant severity-associated factors were identified using a multivariate logistic model and combined to develop a joint index through binary logistic regression analysis. The area under the receiver operating characteristic curve (AUC) was used to identify the optimal model and evaluate the discriminative performance of the index.

**Results:**

In the training cohort, lower CD4+/CD8+ T cell ratio, albumin level, and a higher monocyte percentage (M%) were significant as operating sociated with severe disease (*P* < 0.05 for all). The index was developed using these factors and calculated as CD4+/CD8+ T cell ratio, albumin level, and M%, with a sensitivity and specificity of 90 and 70%, respectively. The AUC values for the index in the training and validation cohorts were 0.85 and 0.75, respectively, indicating good discriminative performance.

**Conclusion:**

We identified disease severity-associated joint index that could be easily evaluated: CD4+/CD8+ T cell ratio, albumin level, and M%.

**Supplementary Information:**

The online version contains supplementary material available at 10.1186/s12888-022-03911-5.

## Introduction

Major Depressive Disorder(MDD) is a prominent public health concern worldwide, and approximately 6% of adults experience MDD per year [1]. The lifetime prevalence of MDD is estimated to be between 2 and 20%, higher than that of other non-communicable diseases [2, 3]. Moreover, MDD is one of the leading causes of disability worldwide [4], with the most dangerous outcome being suicide, which accounts for an estimated 800,000 people a year [5]. Approximately one of every 20 people who attempt suicide die in the attempt. The lifetime prevalence of suicidal ideation is 9.2% [6]. Approximately two-thirds of MDD patients have suicidal thoughts, and up to 15% of MDD patients die by suicide [7]. Clinical factors (especially the severity of depression), previous suicide behaviors, and stressful life events are generally assumed to be the best predictors of suicidal behavior [8]. Thus, more attention should be paid to the assessment of MDD severity.

The etiology and pathogenesis of MDD are complicated and have not been fully elucidated. At present, researchers consider the etiology of MDD to be multifactorial and could be related to genetic, environmental, biological, physiological, psychological, and social factors [9, 10]. Among these factors, inflammatory processes and dysregulation of both the innate and adaptive immune systems are two widely investigated factors that have been considered to be closely related to the pathogenesis of MDD [11]. Moreover, some studies have shown that the severity of depression symptoms and suicide behaviors are positively correlated with inflammation in MDD [12–15]. Thus, inflammatory and immune-related markers from peripheral blood tests may be good indicators of the severity of MDD.

The Third Edition of the American Psychiatric Association (APA) guidelines for the treatment and management of MDD recommended different treatments for depression of mild and moderate and severe (American Psychiatric Association, 2010). Accurate determination of the severity of MDD can help clinicians design appropriate treatment strategies and reduce the disease burden, which can reduce the depression-related suicide rate to some degree [16, 17]. ICD-10 divides depression into mild, moderate, and severe according to the presence of core and non-core symptoms, but this classification method has some limitations, such as an inability to facilitate the evaluation of the influence of suicide and social function impairment on the severity of MDD. In daily clinical practice, two mood screening scales, the Hamilton Depression Scale (HAMD) and Montgomery-Asberg Depression Rating Scale (MADRS), are the most easily understood and commonly used tools for the evaluation of a depressive status and treatment response [18, 19]. Because of the significance accorded to severity by treatment guidelines, it is important to empirically establish cutoff HAMD ratings for the allocation of patients to severity groups. However, some studies on the HAMD and MADRS ratings provided inconsistent results for the cutoff values to define severe depression [20, 21]. Besides, one previous study showed that HAMD had inadequate assessment reliability owing to the poor contribution of several scale items to the measurement of depression severity [22]. Furthermore, the effectiveness of HAMD and MADRS in discriminating the severity of depression remains disputable. Müller et al. [23] showed that MADRS was better than HAMD in terms of discriminating the severity of MDD. On the other hand, Carneiro et al. [24] determined that both scales had adequate reliability and validity for assessing the severity of MDD. Other researchers have used neuroimaging to estimate the clinical changes related to depression [25–28]. Nevertheless, the high costs and prolonged examination durations associated with imaging techniques have limited their application in this context.

Recent research has shown that chronic low-grade inflammation and immune responses play important roles in the disease course of depressive disorder [29, 30]. White blood cells (WBCs), neutrophils, monocytes, lymphocytes and subsets, platelets [31], C-reactive protein (CRP), and albumin can be easily determined from laboratory tests of peripheral blood, which could reflect the systemic inflammatory and immune status. Previous studies have shown that CRP level, mean platelet volume, and the levels of tumor necrosis factor (TNF)-α and cytokines such as interleukin-1 and 6, in peripheral blood were significantly higher in patients with the depressive disorder than in a healthy population [32–35]. Moreover, the proportions of lymphocytes and their subsets significantly decreased in the patients with the depressive disorder compared to that in a healthy population [36–38]. In addition, Shen et al. [39] found that the level of interleukin-18 significantly decreased in patients with MDD who had a HAMD score lower than 7 than in patients with a HAMD score higher than 7 after fluoxetine treatment. This indicates that dynamic change in inflammatory cytokines may reflect the disease severity of MDD [40]. Peripheral blood examinations are more convenient and inexpensive than MRI examinations, and the quality of the results of the assessment is easier to control than the clinical scale. Hence, this study was aimed at determining immune and inflammatory indicators that could be applied to evaluate the severity of depression and developing a novel detection model combining these multiple indicators to classify depression severity.

## Methods and materials

### Participants and study design

This retrospective study was conducted in the Department of Psychiatry, Sir Run Run Shaw Hospital, School of Medicine, Zhejiang University, approved by the ethics committee of the hospital, and performed, according to the Declaration of Helsinki. Patients aged ≥18 years and initially diagnosed with depressive disorder by experienced psychiatrists according to the criteria in the fifth edition of the Diagnostic and Statistical Manual of Mental Disorders (DSM-5) [41] were eligible for inclusion. The exclusion criteria were as follows: (1) a history of hypomanic or manic episodes and a mood disorder questionnaire score of < 7; (2) use of any antidepressants or other antipsychotic medications; (3) presence of comorbidities such as cancer, autoimmune disease, stroke, acute coronary syndrome, and infection; and (4) insufficient liver renal cardiac and bone marrow hematopoietic functions. From August 1, 2020, to December 31, 2020, eligible patients were enrolled into a training cohort. In addition, from January 1, 2021, to April 30, 2021, eligible patients were enrolled in a validation cohort. Simultaneously, a healthy population without any mental disorders was enrolled as a healthy control group to compare their peripheral blood cell and laboratory parameters with those of the MDD patients.

### Disease severity classification

The 24-item HAMD was used to evaluate the status of depressive symptoms in patients through conversation and observation by two well-trained independent investigators. Patients with HAMD scores ranging from 8 to 20 and from 21 to 35 were classified as the mild and moderate depressive disorder groups; respectively, Further patients with HAMD scores of > 35 were classified as the severe depressive disorder group. Patients with mild and moderate depressive disorder were considered the non-severe disease group, and those with severe depressive disorder were considered the severe disease group.

### Data collection

Detailed clinical characteristics and laboratory test data were collected from the electronic medical record of each patient. Baseline laboratory data were collected within 1 week before treatment initiation, including WBC count, absolute neutrophil count (ANC), ANC as a percentage of the WBC count (N%), absolute lymphocyte count (ALC), ALC as a percentage of the WBC count (L%), absolute monocyte count (AMC), AMC as a percentage of the WBC count (M%), platelet count, and serum albumin, CRP and lactate dehydrogenase (LDH) levels. All fasting venous blood samples were collected in the morning and examined by laboratory physicians who were blinded to the clinical status of the study patients.

### Flow cytometry

A 2-mL sample of peripheral blood treated with ethylenediamine tetraacetic acid (EDTA) was obtained from each patient for flow cytometric analysis, and lymphocyte subsets were detected. All tests were performed within 4 h after sampling. To each tube, we added 25 μL of the whole blood sample and 10 μL of mixed antibodies against CD3, CD4, CD8, CD16, CD19, and CD56 (BD Bioscience, Multitest 6-Color TBNK Reagent, 662,967). The tube contents were mixed well, and the tubes were incubated in a dark room at 20–25 °C for 15 min. Then, 450 μL of the erythrocyte lysate was added to each tube and mixed well, and the tubes were incubated in a dark room at a temperature of 20–25 °C for 15 min. After centrifugation at 1500 rpm for 5 min, the supernatant was discarded, and 4–5 drops of sheath solution were added to the machine. Lymphocyte subpopulations were detected by BD Canto II, and data were analyzed by Canto software. The total number of WBCs, percentage of lymphocytes in WBCs, and the absolute number of lymphocytes were detected by blood cell testing with Mindray BC-6900. The relative percentage and absolute count of each lymphocyte subset, including T cells, CD4+ T cells, CD8+ T cells, CD16 + 56+ NK cells, and CD19+ B cells were recorded. The investigators who performed flow cytometry were also blinded to the clinical status of the study patients.

The CD45/SSC gating method was used for lymphocyte subsets, and the specific steps were as follows: ① With CD45 as the horizontal axis and SCC as the vertical axis, CD45/SSClow on the CD45/SSC scatter plot was set as the lymphocyte gate. ② The absolute value and proportion of CD3+ T cells in lymphocytes were analyzed. ③ The absolute value and proportion of CD3 + CD4+ T cells and CD3 + CD8+ T cells were analyzed. ④ The absolute value of CD19+ cells and the absolute value and proportion of B cells and CD16 + 56+ NK cells in lymphocytes were analyzed.

### Statistical analysis

Categorical variables were described as frequencies and percentages, The chi-square test was used to analyze significant differences. The Shapiro-Wilk method was used to test the normality of continuous variables. Continuous variables with normal distribution were described as mean and standard deviation (SD), Student’s t-test was used to analyze significant differences. Continuous variables with a skewed distribution were described as median and interquartile ranges (IQRs), and the Mann-Whitney U test was used to analyze significant differences.

For the training cohort, a univariate logistic regression model was used to select the factors associated with disease severity (*P* < 0.05). These factors were then incorporated into a multivariate logistic regression model to identify significant factors (*P* < 0.05) and calculate odds ratios (ORs) with 95% confidence intervals (CIs) via a forward selection method. Based on independent significant factors and the regression coefficient of each variable, a novel joint index was developed via binary logistic regression analysis to distinguish disease severity. The best cut-off values of the joint index and single significant factors were determined using the maximum Youden index based on the receiver operating characteristic (ROC) curve, which was calculated as the sensitivity plus specificity minus 1. The area under the curve (AUC) method was used to compare the diagnostic efficiency of the joint index and a single significant factor, which was calculated by the 1000 bootstrap resamples method. In addition, the discriminatory performance of the joint index was tested in the validation cohort.

Statistical Product and Service Solutions version 22.0 and the R Programming Language version 3.6.0 were used to perform statistical analysis. All statistical tests were two-tailed and a *P*-value of < 0.05 was considered statistically significant.

## Results

### Demographics and laboratory investigations of patients

From August 1, 2020, to December 31, 2020, 99 patients, including 56 female patients (56.6%) initially diagnosed as showing MDD, were enrolled as the training cohort in this study. Among these, 36, 33, and 30 patients were classified as showing mild, moderate, and severe MDD, respectively. The median age of the patients was 32 years (IQR, 19–48, years). Meanwhile, 38 healthy individuals without any mental disorders were enrolled as healthy control. In the comparison of peripheral blood laboratory parameters, the levels of most immune cells, including CD3+ T cell%, CD3 + CD4+ T cell%, CD4+/CD8+ T cell ratio, CD19+ B cell%, CD16 + 56+ NK cell%, and the CD16 + 56+ NK cell count of the healthy population were higher than that of MDD patients in the training cohort. Detailed clinical characteristics and baseline laboratory investigations of the patients in the training cohort are provided in Table 1.

**Table 1:** Baseline clinical characteristics and laboratory investigation results of patients in the training and validation cohorts and healthy controls

	All patients (***N*** = 196)	Training cohort (***N*** = 99)	Validation cohort (***N*** = 97)	Healthy controls (***N*** = 38)	***P***-value*
**Clinical Characteristics**					
Sex					> 0.99
Female	123 (62.8)	56 (56.6)	67 (69.1)	21 (55.3%)	
Male	73 (37.2)	43 (43.4)	30 (30.9)	17 (44.7%)	
Age, years	32.0 (19.0,51.2)	32.0 (19.0,48.0)	31.0 (19.0,53.0)	34.5 (28.2,51.2)	0.058
Classification					
Mild	67 (34.2)	36 (36.4)	31 (32.0)	0	
Moderate	69 (35.2)	33 (33.3)	36 (37.1)	0	
Severe	60 (30.6)	30 (30.3)	30 (30.9)	0	
**Laboratory Investigation**					
CD3+ T cell%	74.1 (69.3,78.2)	72.7 (67.7,78.0)	76.0 (70.7,78.4)	77.6 (73.8,82.0)	< 0.001
CD3 + CD4+ T cell%	40.6 (±6.89)	39.6 (±6.0)	41.6 (±7.57)	42.3 (37.7,50.3)	0.01
CD3 + CD8+ T cell%	27.6 (24.1,30.7)	27.3 (24.3,30.4)	28.1 (23.8,31.6)	27.9 (24.2,30.8)	0.825
CD4+/CD8+ T cell ratio	1.48 (1.21,1.77)	1.47 (±0.35)	1.47 (1.20,1.91)	1.64 (±0.27)	0.003
CD19+ B cell%	13.6 (10.6,16.5)	13.7 (±5.2)	14.0 (11.2,16.4)	10.8 (9.30,13.0)	0.002
CD16 + 56+ NK cell%	10.2 (6.93,15.0)	11.3 (7.8,17.0)	8.95 (6.48,13.0)	16.0 (11.8,21.4)	0.001
CD3 + CD4 + CD8+ T cell%	0.32 (0.18,0.48)	0.33 (0.16,0.48)	0.31 (0.19,0.48)	0.38 (0.18,0.58)	0.637
CD3+ T cell count, × 10^9^/L	1.44 (1.18,1.73)	1.35 (1.15,1.71)	1.48 (1.23,1.76)	1.41 (1.06,1.71)	0.931
CD3 + CD4+ T cell count, × 10^9^/L	0.78 (0.66,0.96)	0.78 (0.61,0.94)	0.79 (0.67,0.99)	0.71 (0.53,0.91)	0.329
CD3 + CD8+ T cell count, ×10^9^/L	0.54 (0.42,0.70)	0.54 (0.42,0.70)	0.55 (0.42,0.70)	0.52 (0.41,0.67)	0.61
CD3 + CD4 + CD8+ T cell count, × 10^9^/L	0.01 (0.00,0.01)	0.01 (0.00,0.01)	0.01 (0.00,0.01)	0.01 (0.00,0.01)	0.409
CD19+ B cell count, ×10^9^/L	0.25 (0.18,0.37)	0.25 (0.17,0.37)	0.26 (0.20,0.34)	0.20 (0.15,0.27)	0.061
CD16 + 56+ NK cell count, ×10^9^/L	0.19 (0.14,0.30)	0.21 (0.15,0.32)	0.18 (0.12,0.25)	0.29 (0.20,0.39)	0.014
N%	55.7 (±10.1)	56.8 (±9.6)	54.6 (±10.6)	57.3 (±7.60)	0.759
L%	35.0 (28.8,40.2)	33.5 (28.2,39.5)	35.6 (29.5,41.4)	33.8 (26.8,39.6)	0.973
M%	6.25 (5.18,7.30)	6.20 (5.15,7.30)	6.40 (5.20,7.30)	4.96 (4.10,5.35)	< 0.001
WBC count, ×10^9^/L	5.60 (4.77,6.70)	5.60 (4.85,6.80)	5.40 (4.50,6.70)	6.15 (4.93,8.28)	0.067
ANC, × 10^9^/L	3.13 (2.35,3.82)	3.20 (2.63,3.88)	3.05 (2.26,3.77)	3.69 (2.77,4.89)	0.059
ALC, × 10^9^/L	1.89 (1.57,2.36)	1.90 (1.60,2.35)	1.82 (1.56,2.36)	1.75 (1.40,2.35)	0.274
AMC, ×10^9^/L	0.35 (0.28,0.44)	0.35 (0.29,0.44)	0.34 (0.28,0.42)	0.36 (0.28,0.51)	0.572
Platelet count, ×10^9^/L	227 (190,268)	231 (±57.7)	227 (192,269)	241 (±48.4)	0.316
Albumin level, g/L	41.7 (39.7,43.3)	42.2 (±3.3)	41.2 (39.3,42.5)	44.3 (±2.52)	< 0.001
LDH level, U/L	145 (131,166)	148 (130,166)	144 (131,166)	161 (145,177)	0.009
CRP level, mg/L	0.40 (0.20,0.90)	0.50 (0.25,1.10)	0.30 (0.20,0.80)	0.70 (0.40,1.08)	0.115

Data are expressed as N (%) for sex, mean (±standard deviation) for continuous variables with normal distribution, and median (interquartile range) for continuous variables with skewed distribution

* *P*-value of the comparison between the training cohort and healthy controls

Abbreviation: *ANC* absolute neutrophil count, *ALC* absolute lymphocyte count, *AMC* absolute monocyte count, *CRP* C-reactive protein, *L%* lymphocyte count as a percentage of white blood cell count, *M%* monocyte count as a percentage of white blood cell count, *N%* neutrophil count as a percentage of white blood cell count, *LDH* lactate dehydrogenase, *NK* natural killer, *WBC* white blood cell

### Association between disease severity and laboratory parameters

**Fig. 1:**
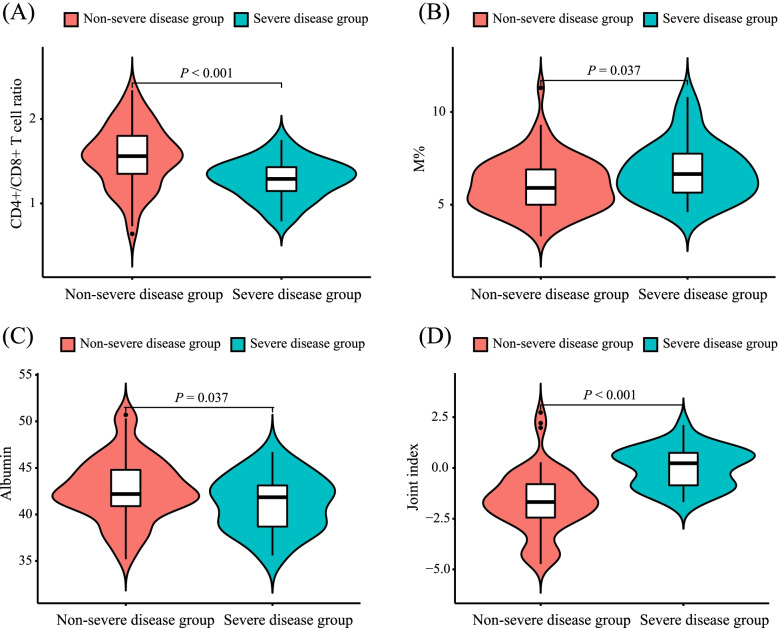
Violin plot for comparing the laboratory parameters between two groups in the training cohort. **A** The mean CD4+/CD8+ T cell ratio in the non-severe disease group was significantly higher than that in the severe disease group (*P* < 0.001); **B** the median M% in the non-severe disease group was significantly lower than that in the severe disease group (*P* = 0.037); **C** the mean albumin concentration in the non-severe disease group was significantly higher than that in the severe disease group (*P* = 0.037); **D** the mean joint index in the non-severe disease group was significantly lower than that in the severe disease group (*P* < 0.001)

In the training cohort, logistic analysis was used to compare the differences in clinical characteristics and laboratory parameters of patients between the non-severe disease group (*n* = 69) and the severe disease group (*n* = 30). A univariate logistic regression model showed that the CD3 + CD4+ T cell% (*P* = 0.033), CD3 + CD8+ T cell% (*P* = 0.007), CD4+/CD8+ T cell ratio (*P* < 0.001; Fig. 1A), M% (*P* = 0.037; Fig. 1B), and albumin level (*P* = 0.037; Fig. 1C) were five factors associated with disease severity, while the groups showed no differences in age, sex, and other laboratory parameters (Table 2).

**Table 2:** Univariate logistic regression analysis of MDD severity in the training cohort

	Non-severe group (***N*** = 69)	Severe group (***N*** = 30)	Statistical magnitude	***P***-value
Sex			0.802 ^a^	0.37
Female	37 (53.6)	19 (63.3)		
Male	32 (46.4)	11 (36.7)		
Age, years	32.0 (18.0,48.0)	27.5 (19.0,40.2)	−0.377 ^c^	0.706
CD3+ T cell%	72.0 (67.7,76.7)	74.2 (69.4,79.3)	−1.211^c^	0.226
CD3 + CD4+ T cell%	40.4 (±6.13)	37.6 (±5.37)	2.161 ^b^	0.033
CD3 + CD8+ T cell%	26.3 (22.9,29.8)	29.5 (26.6,32.0)	−2.695 ^c^	0.007
CD4+/CD8+ T cell ratio	1.55 (±0.37)	1.28 (±0.23)	4.481 ^b^	< 0.001
CD19+ B cell%	13.8 (±5.09)	13.5 (±5.55)	0.303 ^b^	0.763
CD16 + 56+ NK cell%	12.1 (7.75,17.3)	11.0 (7.75,14.0)	−0.857 ^c^	0.392
CD3+ T cell count, ×10^9^/L	1.40 (1.15,1.70)	1.33 (1.19,1.71)	−0.423 ^c^	0.673
CD3 + CD4+ T cell count, × 10^9^/L	0.81 (0.66,0.94)	0.72 (0.59,0.87)	−1.687 ^c^	0.092
CD3 + CD8+ T cell count, ×10^9^/L	0.53 (0.42,0.71)	0.54 (0.44,0.70)	−0.438 ^c^	0.661
CD3 + CD4 + CD8+ T cell count, ×10^9^/L	0.01 (0.00,0.01)	0.01 (0.00,0.01)	−0.441 ^c^	0.659
CD19+ B cell count, ×10^9^/L	0.26 (0.18,0.37)	0.24 (0.16,0.39)	−0.465 ^c^	0.642
CD16 + 56+ NK cell count, ×10^9^/L	0.22 (0.16,0.36)	0.19 (0.15,0.25)	−1.425 ^c^	0.154
N%	57.4 (±9.79)	55.3 (±9.24)	1.022 ^b^	0.309
L%	32.8 (28.0,39.5)	36.1 (29.1,40.6)	−0.845 ^c^	0.398
M%	5.90 (5.00,6.90)	6.65 (5.65,7.75)	−2.083 ^c^	0.037
WBC, ×10^9^/L	5.60 (5.10,6.90)	5.30 (4.70,6.45)	−1.436 ^c^	0.151
ANC, ×10^9^/L	3.33 (2.83,3.96)	2.83 (2.23,3.70)	−1.934 ^c^	0.053
ALC, ×10^9^/L	1.90 (1.60,2.40)	1.80 (1.52,2.28)	−0.618 ^c^	0.537
AMC, ×10^9^/L	0.36 (0.29,0.43)	0.35 (0.29,0.49)	−0.636 ^c^	0.525
Platelet, ×10^9^/L	226 (±52.2)	243 (±68.3)	−1.351 ^b^	0.18
Albumin, g/L	42.7 (±3.38)	41.2 (±2.93)	2.112 ^b^	0.037
LDH, U/L	147 (132,164)	148 (130,166)	−0.19 ^c^	0.849
CRP, mg/L	0.60 (0.30,1.10)	0.35 (0.23,0.60)	−1.017 ^c^	0.309

^a^ Chi-square value for chi-square test; ^b^ t value for t-test; ^c^ Z value for Mann-Whitney *U* test; * multiple *P*-value comparisons via Benjamini-Hochberg method

Abbreviations: *ANC* absolute neutrophil count, *ALC* absolute lymphocyte count, *AMC* absolute monocyte count, *CRP* C-reactive protein, *L%* lymphocyte count as a percentage of white blood cell count, *M%* monocyte count as a percentage of white blood cell count, *N%* neutrophil count as a percentage of white blood cell count, *LDH* lactate dehydrogenase, *NK* natural killer, *WBC* white blood cell

The multivariate logistic regression model showed that the mean CD4+/CD8+ T cell ratio of the non-severe disease group was significantly higher than that of the severe disease group (1.55 vs. 1.28; OR 0.019; 95% CI: 0.003–0.140; *P* < 0.001); the mean albumin level of the non-severe disease group was significantly higher than that of the severe disease group (42.7 vs. 41.2 g/L; OR 0.839; 95% CI: 0.708–0.994; *P* = 0.043); and the median M% of the non-severe disease group was significantly lower than that of severe disease group (5.90 vs. 6.65; OR 1.686; 95% CI: 1.139–2.494; *P* = 0.009; Table 3).

**Table 3:** Multivariate logistic regression analysis of MDD severity in the training cohort

	β-coefficient	Wald	OR (95% CI)	***P***-value
CD4+/CD8+ T cell ratio	−3.959	15.131	0.019 (0.003,0.140)	< 0.001
Albumin	−0.175	4.103	0.839 (0.708,0.994)	0.043
M%	0.522	6.829	1.686 (1.139,2.494)	0.009

Abbreviations: *M%* monocyte count as a percentage of white blood cell count

### Construction and evaluation of the novel joint index

Based on the three independent significant factors, namely, CD4+/CD8+ T cell ratio, albumin level, and M%, a novel joint index was constructed through binary logistic regression analysis. The formula of the joint index was as follows:

$$\mathrm{Joint}\ \mathrm{index}=-3.959\times \mathrm{CD}4+/\mathrm{CD}8+\mathrm{T}\ \mathrm{cell}\ \mathrm{ratio}-0.175\times \mathrm{albumin}+0.522\times \mathrm{M}\%+8.739$$

**Fig. 2:**
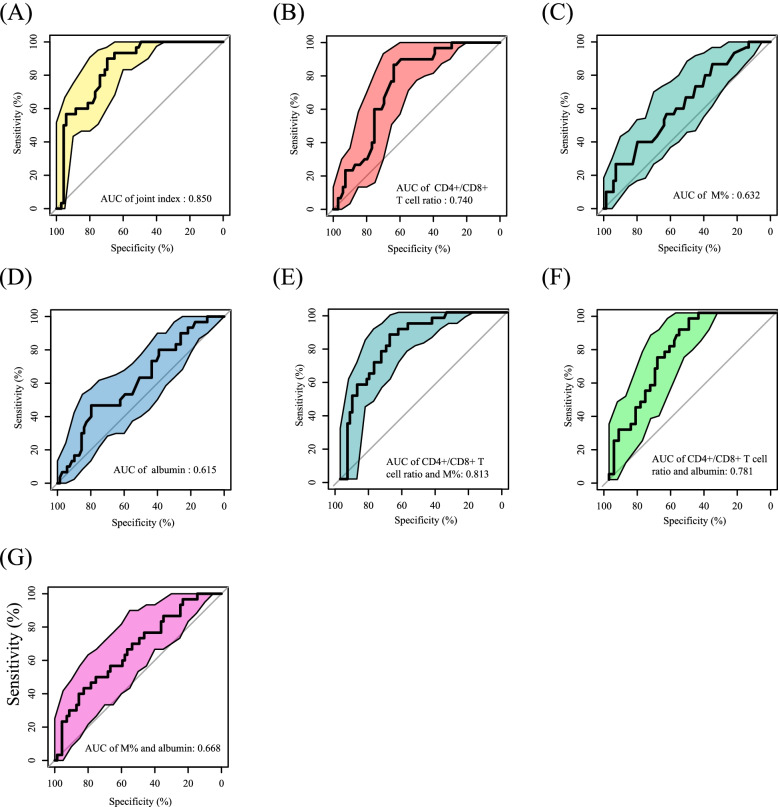
ROC curves for the joint index, single variables, and two-variable combinations for the discrimination of disease severity in the training cohort. **A** The AUC value of the joint index is 0.850 (95% CI: 0.774–0925); **B** The AUC value of the CD4+/CD8+ T cell ratio is 0.740 (95% CI: 0.643–0.837); **C** The AUC value of the M% is 0.632 (95% CI: 0.516–0.749); **D** The AUC value of the albumin level is 0.615 (95% CI: 0.496–0.734). **E** The AUC value of the CD4+/CD8+ T cell ratio and M% combination is 0.813 (95% CI: 0.734–0.895); **F** The AUC value of the CD4+/CD8+ T cell ratio and albumin level combination is 0.781 (95% CI: 0.693–0.869); **G** The AUC value of the albumin level and M% combination is 0.668 (95% CI: 0.553–0.782)

The logistic regression model showed that the mean joint index in the non-severe disease group was significantly lower than that in the severe disease group (− 1.71 [±1.52] vs. 0.01 [±0.96]; *P* < 0.001; Fig. 1D). The ROC curves suggested that the best cut-off value of the joint index was − 1.2, indicating that patients with joint index values higher than − 1.2 had a higher probability of showing severe MDD, while those with joint index values less than − 1.2 had a higher probability of showing mild or moderate MDD. The AUC of the joint index was 0.850 (95% CI: 0.774–0.925), and the sensitivity and specificity were 90.9 and 70.0% respectively (Fig. 2A). Meanwhile, the best cut-off values of the CD4+/CD8+ T cell ratio, albumin level, and M% were 1.5, 40.6, and 5.3, respectively. The corresponding AUC values were 0.740 (95% CI: 0.643–0.837), 0.615 (95% CI: 0.496–0.734), and 0.632 (95% CI: 0.516–0.749) (Fig. 2B-D), which were all significantly lower than that of the joint index (all *P* < 0.05; Table 4). We also evaluated the performance of different combinations of two significant variables and found that the AUCs of three different combinations were all significantly lower than that of the joint index (Fig. 2E-G and Supplementary Materials, Table S1).

**Table 4:** Comparison of the discriminative performance between the joint index and individual factors in the training cohort

	Best cut-off value	AUC (95% CI)	Sensitivity (%)	Specificity (%)	***P***-value
Joint index	−1.2	0.850 (0.774–0.925)	90.0	70.0	Reference
CD4+/CD8 + T cell ratio	1.5	0.740 (0.643–0.837)	86.7	63.8	0.015
M%	5.3	0.632 (0.516–0.749)	86.7	34.8	< 0.001
Albumin level	40.6	0.615 (0.496–0.734)	46.7	79.7	< 0.001

Abbreviations: *M%* monocyte count as a percentage of the white blood cell count

### Discriminative performance of the joint index in the validation cohort

From January 1, 2021, to April 30, 2021, 97 MDD patients were enrolled as a validation cohort of which 31, 36, and 30 patients were classified as showing mild-moderate, and severe MDD, respectively. The other detailed clinical characteristics and laboratory investigation of patients in this cohort are shown in Table 1.

In the validation cohort, the AUC of the joint index was 0.750 (95% CI: 0.645–0.849), and the sensitivity and the specificity were 86.7 and 62.7% respectively (Supplementary Materials, Fig. S2A). Moreover, the median joint index value of the non-severe disease group was also significantly lower than that of the severe disease group (− 1.76 [IQR -3.29, − 0.46] vs. 0.03 [IQR -0.73, 1.43]; *P* < 0.001; Supplementary Materials, Fig. S2B and Table S2).

## Discussion

This research identified the CD4+/CD8+ T cell ratio, albumin level, and M% as three laboratory parameters significantly associated with the severity of MDD. Based on these three factors, we constructed a novel joint index that showed a good performance in discriminating disease severity, and we validated our findings in the validation cohort. Moreover, the establishment of this index could provide useful laboratory indicators for clinicians to evaluate the severity of depression, and thus provide an effective method for the establishment of clinical treatment and nursing decision-making. Nevertheless, given that the sample size of this study is too small, the efficiency of the model can be improved if the sample size can be increased. In our study, an increase in the CD4+/CD8+ T cell ratio, M%, and albumin level was observed, single variables (CD4+/CD8+ T cell ratio, M%, and albumin level) and two-variable combinations (CD4+/CD8+ T cell ratio and M%, CD4+/CD8+ T cell ratio and albumin level, and albumin level and M% exhibited a low AUC for predicting the severity of depression in comparison with the three-variable combination (joint index). However, combining the three ratios resulted in an AUC of 0.75, suggesting that the combination was a powerful marker for predicting the severity of depression.

In the present study, we found that the CD4+/CD8+ T cell ratio and albumin concentration of patients with mild and moderate depressive disorder were significantly higher than those of patients with severe depressive disorder, while the M% of patients with the mild and moderate depressive disorder was significantly lower than that of patients with MDD. Several studies have demonstrated that changes in immune functions may play an important role in the disease process underlying depressive disorders [42]. A literature review showed that T cells and NK cells became more active as depressive symptoms improved after antidepressant treatment [43, 44]. Moreover, another study suggested that antidepressant treatments contributed to an upregulation of Tregs in MDD patients [45]. Miller suggested that Tregs may contribute to the severity of depression through downregulation of the chronic inflammatory response [46]. Jha et al. [47] found that adolescent and young adult patients with recent suicide behavior exhibit lower IL-4 levels, and reduced IL-4 levels may indicate an increased risk of autoimmunity. Dysregulation of the hypothalamic-pituitary-adrenal axis is a hallmark of depressive disorder, which results in the hypersecretion of cortisol and exerts inhibitory effects on the immune system by suppressing the cellular immune response and increasing inflammatory cytokines [48] A previous study investigated the specific alterations in the lymphocyte subsets of patients with MDD and found that the level of CD8+ T cells in these patients was higher than that in the healthy control population [49]. In addition, another study showed that cortisol could increase the concentration of serum soluble CD8 or suppressor/cytotoxic antigen to suppress the immune response of patients with MDD [50]. The levels of depressive symptoms are not associated with serum cortisol concentration, but our study design limited our ability to discriminate because the sample size was restricted to 12 for study eligibility, limiting our ability to assess the association with the severity of depressive symptoms. However, in this study, among 12 patients with serum cortisol concentration data, we did not find significant correlations with several immune cells, including CD3+ T cell count, CD3 + CD4+ T cell count, CD3 + CD8+ T cell count, CD19+ B cell count, and ALC (Table S3 and Fig. S1). These results indicated that depressive disorder may damage the immune system and result in immunosuppression. In this study, we found that the CD4+/CD8+ T cell ratio of patients with MDD was significantly lower, indicating an immune function disorder.

Albumin, the most important protein in human plasma, is mainly produced by the liver, and it reflects the body’s nutritional balance and helps maintain its osmotic pressure. Hypoalbuminemia has been reported in patients with mood disorders in several previous studies [51–54]. In addition, a diet-controlled study also demonstrated that the serum albumin level was significantly lower in patients with depressive disorders than in normal volunteers and that the reduced albumin level was related to the severity of the disease when rated by the HAMD score, which was consistent with the results of this study [55]. Psychiatric illness may influence the serum concentrations of albumin by altering daily behaviors such as ingestion; thus, hypoalbuminemia in patients with depressive disorders may be due to dietary deficiencies. Serum albumin is a routinely tested factor from peripheral blood and can be considered as an easily available biomarker for accurate classification of the severity of the depressive disorder.

Monocytes originate from their progenitors in the bone marrow and are transported to the peripheral blood via the bloodstream. During inflammation, circulating monocytes are recruited by a series of chemokines and migrate into tissues, where they differentiate into macrophages or dendritic cells after conditioning by pro-inflammatory cytokines and microbial products. This process is essential for the effective control of infection and is involved in the pathogenesis of inflammatory and degenerative diseases [56]. A previous study found that the monocyte count and monocyte-to-lymphocyte ratio were significantly higher in patients with MDD than in healthy controls [57]. Similarly, another study found that patients with MDD exhibited significantly higher levels of serum pro-inflammatory IL-12 and IL-6, which were associated with increased numbers of circulating non-classical CD11b^+^CD16^+^CD14^+^ monocytes and an increased activation state of classical CD40^+^CD86^+^ monocytes [58]. These findings were also consistent with the results of the present study, which showed that the peripheral blood M% was higher in patients with depressive disorder patients with severe disease, indicating the potential diagnostic value of monocyte counts.

Notably, a novel joint index based on the CD4+/CD8+ T cell ratio, albumin concentration, and M% was developed in the present study, and it showed superior sensitivity and specificity to discriminate the severity of the depressive disorder in comparison to the individual significant factors. Moreover, the joint index score also showed good discriminative performance in the validation cohort. This novel joint index was based on objective biological markers from peripheral blood, which could facilitate a more easy and accurate severity classification of depressive disorder.

## Limitations

This study had a few limitations. First, the healthy control population was small. Second, the sample size was relatively small, even if this study populations include validation and control groups. It is much better to collect more samples and conduct an external validation in the future. Eventually, conclusive evidence will be more concrete and feasible in clinical practice and a prospective study with a larger study population was required to verify the efficacy of the joint index. Finally, this study focused on treatment-naïve patients and did not analyze the association between variations in laboratory parameters after subsequent therapy and changes in the depressive disorder status.

## Conclusion

In conclusion, we identified three laboratory parameters, namely CD4+/CD8+ T cell ratio, albumin concentration, and M%, that were associated with the severity of the depressive disorder, and constructed a novel joint index to discriminate disease severity more objectively and sensitively.

## Supplementary Information

**Additional file 1.**

## Data Availability

The datasets generated and analyzed during the current study are available from the corresponding author on reasonable request.

## Abbreviations

MDD: Major depressive disorder
HAMD: Hamilton Depression Scale
MADRS: Montgomery-Asberg Depression Rating Scale
CRP: C-reactive protein
TNF: Tumor necrosis factor
DSM-5: A diagnostic and statistical manual of mental disorders
WBC: White blood cell
ANC: Absolute neutrophil count
LDH: Lactate dehydrogenase
EDTA: Ethylene diamine tetraacetic acid
SD: Standard deviation
IQR: Interquartile ranges
OR: Odds ratio
CI: Confidence intervals
ROC: Receiver operating characteristic
AUC: Area under the curve
