# Supplementary material for: A novel joint index based on peripheral blood CD4+/CD8+ T cell ratio, albumin level, and monocyte count to determine the severity of major depressive disorder
Source: BMC Psychiatry. 2022 Apr 8;22:248. doi: 10.1186/s12888-022-03911-5 (PMC8991892; doi:10.1186/s12888-022-03911-5)
Supplement: Supplementary file 1 — Additional file 1. [file 12888_2022_3911_MOESM1_ESM.docx]

Table S1. Comparison of the discriminative performance between the joint index, single variables, and two-variable combinations in the training cohort

| Variable |  | AUC (95% CI) | *P*-value |
| --- | --- | --- | --- |
| Three-variable combination | Joint index | 0.850 (0.774-0.925) | reference |
| Single variable | CD4+/CD8+ T cell ratio | 0.740 (0.643-0.837) | 0.015 |
|  | M% | 0.632 (0.516-0.749) | <0.001 |
|  | Albumin level | 0.615 (0.496-0.734) | <0.001 |
| Two-variable combination | CD4+/CD8+ T cell ratio and M% | 0.813 (0.734-0.895) | 0.031 |
|  | CD4+/CD8+ T cell ratio and albumin level | 0.781 (0.693-0.869) | 0.02 |
|  | Albumin level and M% | 0.668 (0.553-0.782) | <0.001 |

Table S2. The statistical parameters for the difference between the joint index of the severe and non-severe groups in the training and validation cohorts.

|  | All patients | Non-severe group | Severe group | t-value | *P*-value |
| --- | --- | --- | --- | --- | --- |
| Training cohort | -1.1868 (1.59) | -1.7085 (1.52) | 0.0131 (0.96) | -4.398 | <0.001 |
|  |  |  |  | Z-value |  |
| Validation cohort | -1.05 (-2.96, 0.23) | -1.76 (-3.29, -0.46) | 0.03 (-0.73, 1.43) | -5.505 | <0.001 |

Table S3. Spearman correlation analysis for serum cortisol level and immune cell counts

| Spearman correlation analysis | | CD3+ T cell count | CD3+CD4+ T cell count | CD3+CD8+ T cell count | CD19+ B cell count | ALC |
| --- | --- | --- | --- | --- | --- | --- |
| Serum cortisol level | Correlation coefficient | 0.53 | 0.51 | 0.30 | 0.41 | 0.45 |
|  | *P*-value | 0.07 | 0.09 | 0.35 | 0.18 | 0.14 |

Figure S1. Spearman correlation analysis of serum cortisol level and immune cell counts


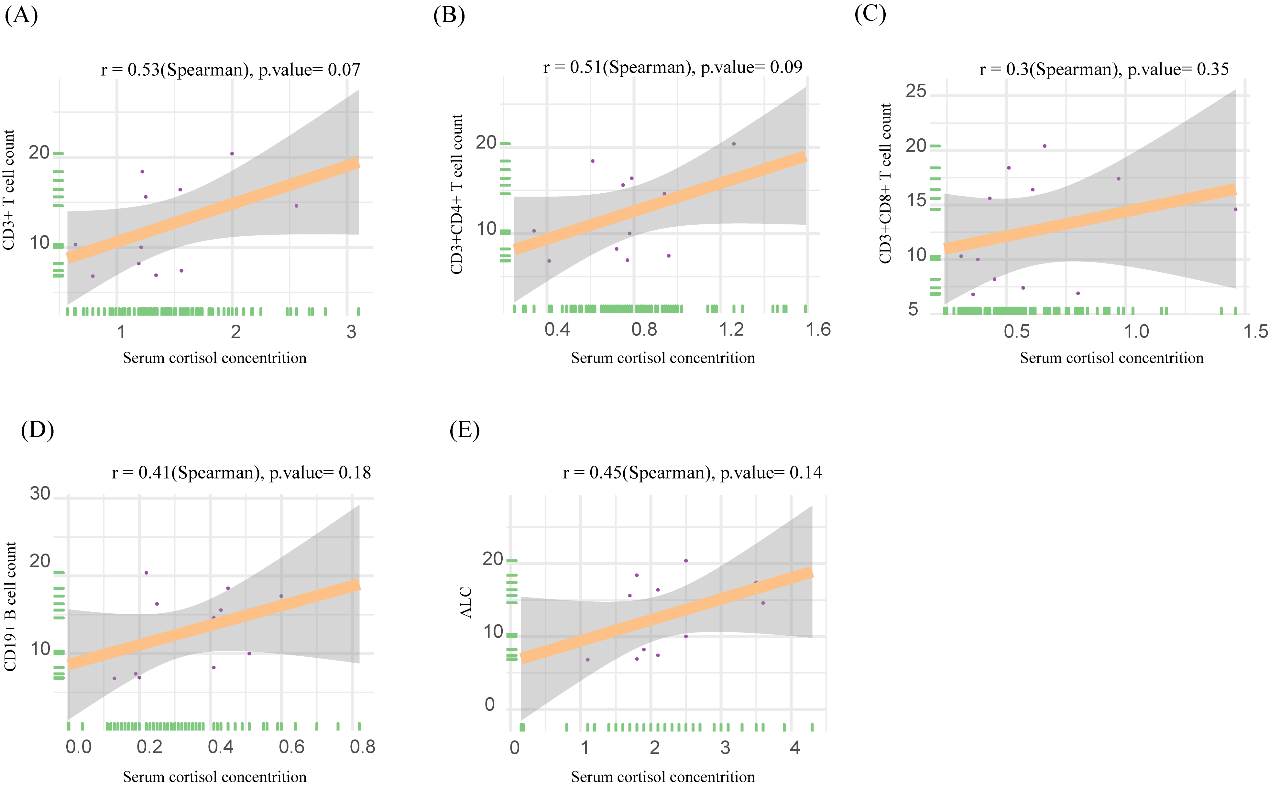


1. The coefficient for the correlation between serum cortisol level and CD3+ T cell count (r = 0.53, P-value = 0.07);
2. The coefficient for the correlation between serum cortisol level and CD3+CD4+ T cell count (r = 0.51, P-value = 0.09);
3. The coefficient for the correlation between serum cortisol level and CD3+CD8+ T cell count (r = 0.30, P-value = 0.35);
4. The coefficient for the correlation between serum cortisol level and CD19+ B cell count (r = 0.41, P-value = 0.18);
5. The coefficient for the correlation between serum cortisol level and ALC (r = 0.45, P-value = 0.14).

Figure S2. ROC curves and violin plot of the joint index to discriminate disease severity in validation cohort


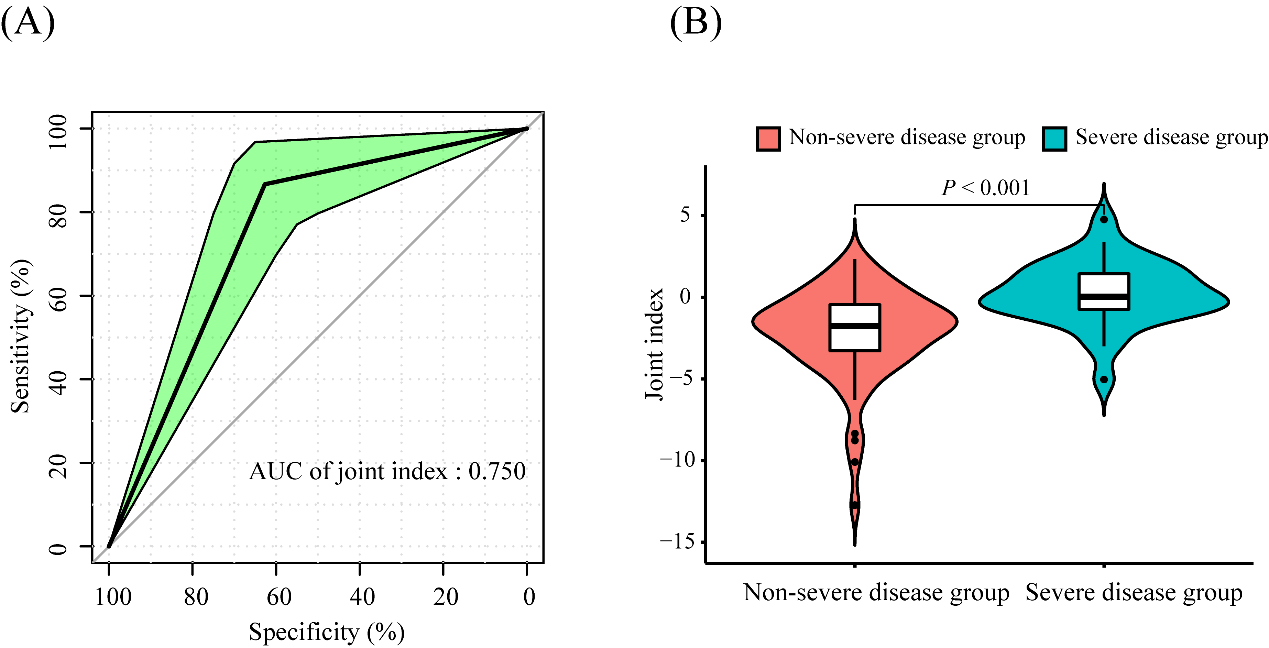


(A) The AUC of the joint index is 0.750 (95% CI: 0.645-0.849); (B) the median joint index in the non-severe disease group was also significantly lower than in the severe disease group (*P* < 0.001).
